# Analysis of Digital Workflow in Implantology

**DOI:** 10.1155/2021/6655908

**Published:** 2021-02-15

**Authors:** Gustavo Vargas da Silva Salomão, Eliseo Pablo Chun, Renato dos Santos Panegaci, Fernando Toledo Santos

**Affiliations:** ^1^Department of Oral Rehabilitation, School of Dentistry, Ibirapuera University (Unib), Av. Interlagos, 1329, Chácara Flora, São Paulo, SP 04661-100, Brazil; ^2^Department of Prosthodontics, School of Dentistry, Ibirapuera University (Unib), Av. Interlagos, 1329, Chácara Flora, São Paulo, SP 04661-100, Brazil

## Abstract

Digital workflow is increasingly accessible in daily dental practice. It has several benefits in implantology, such as the possibility of precise planning, which results in faster and safer surgery and, consequently, reduced prosthetic complications. There are also disadvantages that must be taken into consideration for successful treatment, such as deviations between the planned and placed implant position and intraoral scanning inaccuracies. We report a clinical case in implantology in which digital workflow was used throughout the process, pointing out its facilities and complications in the daily practice of dental surgeons. The patient had grade II mobility and external root resorption of tooth 11. After virtual planning, a surgical guide was fabricated by a CAD/CAM system, with immediate placement of a dental implant using the guided surgery technique. At the end of the osseointegration period, intraoral scanning was performed for fabrication of the final prosthesis also by a CAD/CAM system. After placement, the patient approved the aesthetic and functional results of the implant. We observed advantages such as simplification of clinical steps and safety of the proposed planning, but there were also disadvantages such as the complexity of digital tools, deviations of the placed implant, and inaccuracy in color selection. It was concluded that digital workflow is a reality that can be integrated into daily dental practice, resulting in greater safety, predictability of results, and ease of use in all clinical stages. However, it should be noted that there are still inaccuracies in digital tools and that a steep learning curve is needed in this area, which, if neglected, may lead to unsatisfactory results.

## 1. Introduction

Digital workflow in dentistry has increased rapidly in recent years due to advances in technologies such as software and intraoral scanners [1, 2]. This evolution resulted in benefits such as predictability of treatment, simplification of clinical steps, and quick and easy communication between professionals [3, 4]. The possibilities of treatment involving digital workflow have also evolved with new materials and techniques. The fabrication of laboratory works by a computer-aided design (CAD) and computer-assisted manufacturing (CAM) system associated with several digital tools has allowed a range of options for the proposed work [5].

In implantology, digital workflow has several advantages, such as precise planning and simplified surgical approach, which result in a faster and more predictable surgery [6]. At the prosthesis manufacturing stage, it is possible to reproduce the implant position virtually by intraoral scanning [7], thus avoiding distortions generated by conventional impression materials, promoting greater patient comfort, and eliminating the need for plaster dental casts [8, 9].

Digital techniques also have disadvantages, such as deviations between the planned and placed implant position [10] and inaccuracies of the intraoral scanning technique [11, 12]. These factors are extremely important for clinical treatment because, if they are not taken into account during planning, undesirable results may occur, such as invasion of the space of noble areas and misfit of prosthetic components.

Although apparently easy to perform, digital workflow requires extensive experience of the operator, as it can produce unsatisfactory results if not performed accurately and appropriately [13, 14]. Digital treatment options do not obviate the need for theoretical knowledge. Therefore, the clinician needs to have both a conceptual understanding of the procedures to be performed and the ability to use digital tools to achieve the expected success [13].

The present clinical case report has the novel purpose of demonstrating all clinical stages of digital workflow in implantology, pointing out its facilities and complications in the daily practice of dental surgeons.

## 2. Case Presentation

This case was reported according to the Case REport (CARE) statement and checklist [15], and written informed consent was obtained from the patient to publication of this case report.

A 29-year-old female patient, nonsmoker, who reported not consuming alcoholic beverages, not taking any long-term medication, and having no clinically relevant family history information, complained of pain on touching the maxillary left central incisor (tooth 11). Her dental history included injury to the tooth in question due to a fall from standing height during childhood, and the tooth began to be dislodged over the years. In a specialized dental care clinic, she received endodontic treatment that did not achieve the expected success.

**Figure 1:**
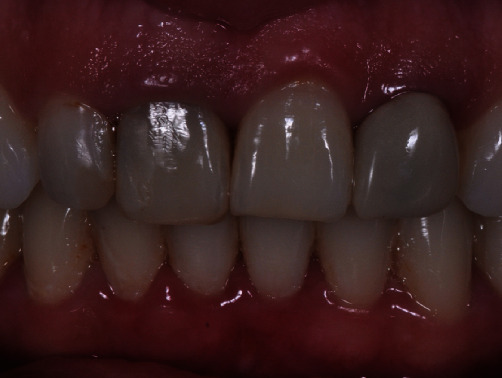
Initial clinical image of the case.

**Figure 2:**
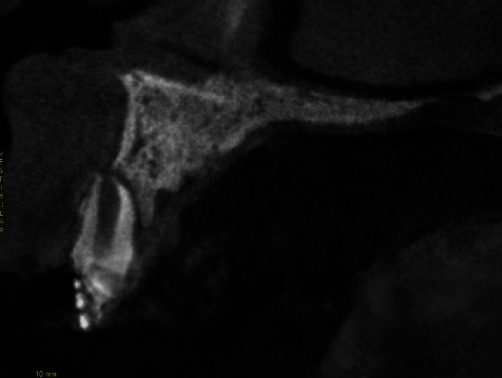
CT scan of tooth 11 area, where it is possible to note external root resorption and extensive resorption of the buccal wall.

Clinical examination showed grade II mobility of tooth 11. Radiographic examination revealed the presence of external root resorption that resulted in extensive resorption of the buccal wall in the area (Figures 1 and 2).

After evaluating the case, extraction of tooth 11 was proposed to the patient, followed by immediate placement of a dental implant associated with guided bone regeneration, because her gingival biotype was classified as thick [16], and the remaining bone tissue allowed immediate implant placement [17]. The management option of choice was a fully digital treatment, from surgical planning to fabrication of the final crown, for the current analysis of digital workflow in dentistry.

A computed tomography (CT) scan of the complete maxilla was obtained in Digital Imaging and Communications in Medicine (DICOM) format, and intraoral scanning was performed using the Trios 3® pod scanner (3shape) to obtain stereolithography (STL) files.

**Figure 3:**
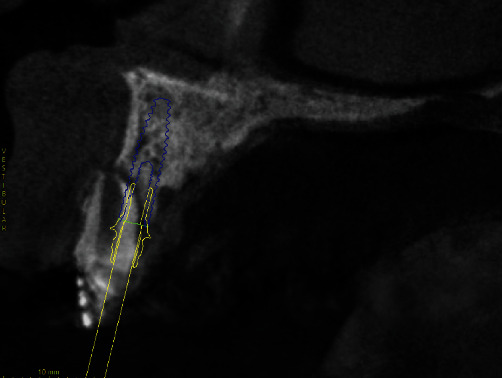
Virtual implant placement in the optimal position for future prosthetic rehabilitation.

**Figure 4:**
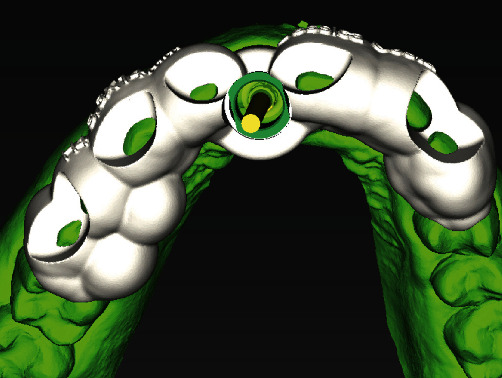
Image of the surgical guide virtually designed using virtual planning software.

Once these 2 files had been obtained, the coDiagnostiX® software (Dental Wings) was used for virtual planning. After DICOM and STL images were superimposed, surgical planning was performed by placing the implant in the optimal position for future prosthetic rehabilitation (Figure 3). Once the implant had been virtually planned, a software for manipulation of 3D objects (Meshmixer®) was used to remove tooth 11 from the original STL file in order to fabricate a tooth-supported surgical guide. This new STL file was imported, and the process of guide fabrication was started by using the “Add surgical guide” tool in coDiagnostiX®. The final virtually designed guide (Figure 4) was then printed with a Rapid Shape®-Straumann 3D printer.

**Figure 5:**
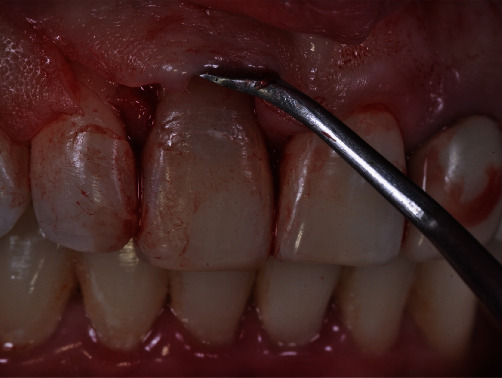
Incision made prior to extraction. Note that the papillae were preserved.

**Figure 6:**
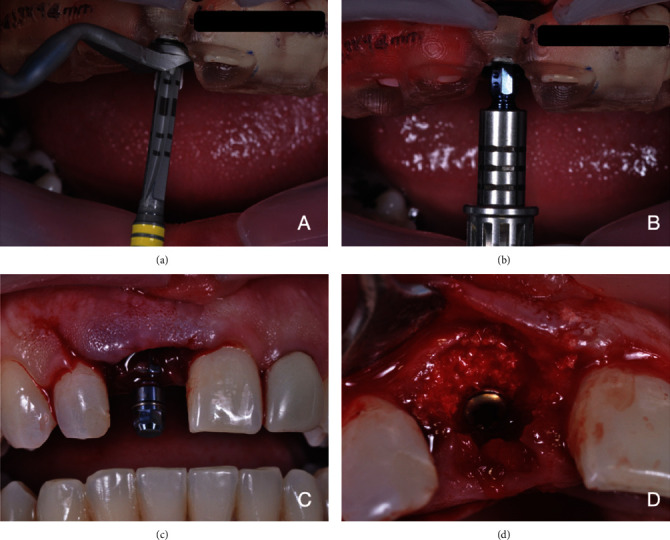
Presentation of the guided implant placement surgery: (a) implant bed preparation; (b) fully guided implant placement; (c) checking the implant for proper final position in relation to the adjacent teeth; (d) guided bone regeneration after implant placement.

At the time of surgery, before the sterile drapes were applied, intraoral antisepsis was performed by mouth rinses with 5 mL of 0.12% aqueous chlorhexidine, and extraoral antisepsis was performed with 2% aqueous chlorhexidine. Anesthesia was obtained with 4% articaine with 1 : 100,000 epinephrine (4% Articaine DFL®). Initially, a buccal incision was made 2 mm above the mesial papillae (from tooth 21 to 12), in order to have them preserved, followed by a divergent relaxing incision in the mesial side of tooth 12 extending to the mucogingival line [18] (Figure 5). The flap was then elevated, and tooth 11 was extracted using a minimally invasive approach with periotomes (Quinelato). After extraction, the surgical guide was placed in position to assess proper fitting through the inspection windows. After this stage, the implant bed was prepared with drills from the Straumann ® Guided Surgery–BLT system, as described in the protocol printed by the planning software (Figure 6(a)). Subsequently, a 3.3 × 14 mm Straumann SLActive® Bone Level Tapered Roxolid implant (NC) was placed, reaching a maximum torque of 45 Ncm (Figures 6(b) and 6(c)). Its respective tap was then inserted. Particulate bone substitute (Geistlich Bio-oss**®**) was placed on the exposed threads, as planned virtually, for the purpose of guided bone regeneration [19, 20] (Figure 6(d)). Once bone grafting had been completed, the flap was repositioned and secured with simple interrupted sutures (4-0 silk suture, Ethicon) for healing by first intention. Postoperative care included administration of antibiotics (amoxicillin 500 mg, every 8 hours for 7 days), anti-inflammatory drugs (dexamethasone 4 mg, every 12 hours for 2 days), and analgesics (acetaminophen 750 mg, every 6 hours for 3 days in case of pain).

The crown of tooth 11 was prepared and temporarily bonded with light-cured resin (Tetric N-Ceram®–Ivoclar Vivadent) to the adjacent teeth (teeth 21 and 12) until the time of implant uncovering.

**Figure 7:**
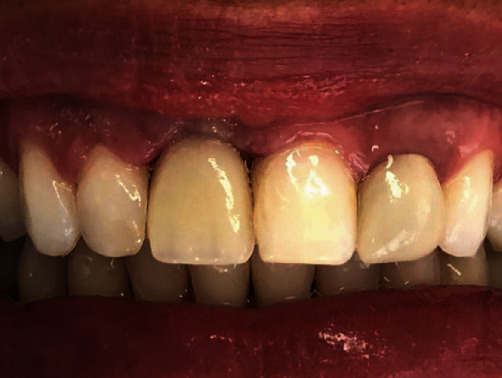
Placement of the temporary abutment on the implant, after implant uncovering.

After 45 days, the implant was uncovered. The surgical guide was again positioned in the mouth, and using a 3.4 mm diameter mucosa punch (Straumann Guided Surgery®–BLT), the gingival tissue was removed, and the tap was exposed. A temporary abutment for NC–BLT implant (Straumann) was then placed, while the provisional crown was captured in the mouth (artificial teeth–Trilux–EuroVIPI). After polishing and finishing, the temporary abutment was tightened with manual torque (Figure 7).

**Figure 8:**
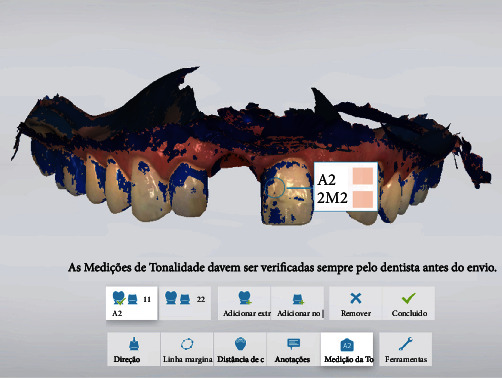
Selection of the final crown shade using the intraoral scanner.

The patient was scheduled to return in 4 weeks for evaluation of the peri-implant tissue. At this visit, minor adjustments were also made to the crown to improve the gingival contour. The patient was then scheduled to return in 6 months for fabrication of the final prosthesis. At this return visit, the provisional crown was removed, and a scanbody (NC Straumann) was placed. After checking the scanbody for proper position, another intraoral scanning was performed (Trios 3® pod–3shape) for fabrication of the final prosthesis. During scanning, the patient's tooth shade was determined by using the scanner's shade measurement tool (Figure 8) and the VITA Classical shade guide. Subsequently, both the STL files and the visually selected shades were sent to the laboratory for fabrication of the final prosthesis.

Given the precision of the digital workflow, the final abutment (3.8 mm diameter, 5.5 mm height, 1 mm gingival height; NC–Variobase®), preselected during virtual planning, was sent to the laboratory, which was requested to send us the final crown, thus obviating the need for an appointment for metal framework try-in. A specific CAD dental software (Dental System–3shape®) was used to design the crown, which was subsequently milled with the Ceramill® Motion 2 milling machine (Amann Girrbach) (CAM system). On delivery, minor adjustments were made to the prosthesis to eliminate occlusal interference. After polishing and patient's approval of aesthetics and function, the crown was placed on the implant with a torque of 35 Ncm.

**Figure 9:**
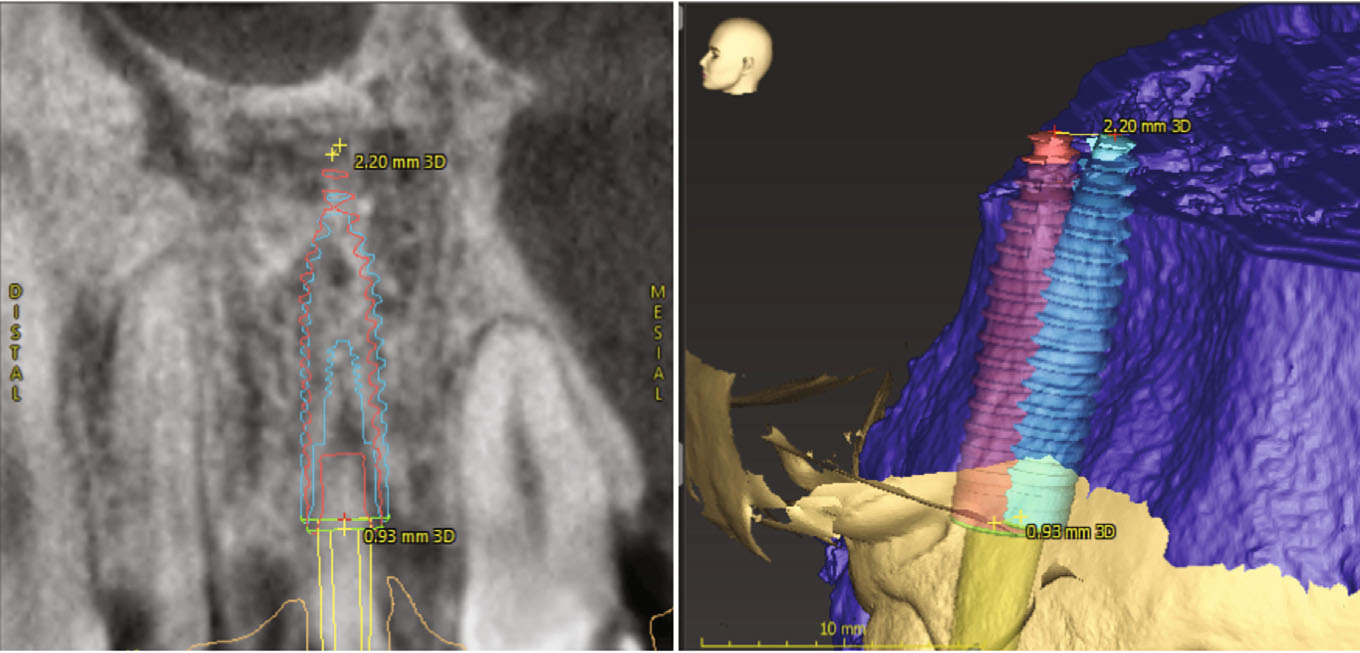
Deviations observed in superimposed images of the placed and planned implant position in the tangential and axial views, respectively.

The coDiagnostiX® “treatment evaluation” tool was used to assess how much the implant deviated from the preoperatively planned position. The STL image produced by intraoral scanning through the scanbody was superimposed onto the software planned implant position, thus generating the deviations (Figure 9).

**Figure 10:**
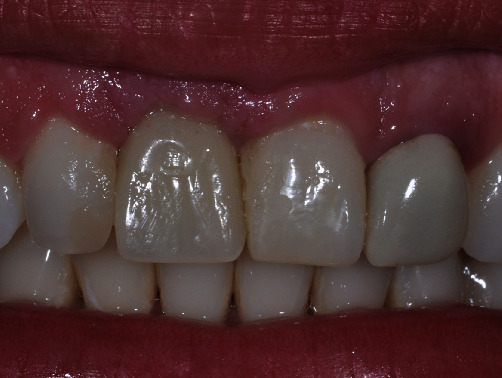
Aesthetic result 3 months after final crown placement.

The patient returned for treatment evaluation 3 months after crown delivery, when she reported satisfaction with the aesthetic and functional results of the final crown (Figure 10), without any complications since its placement.

## 3. Discussion

Digital workflow is constantly and rapidly evolving in implant dentistry, resulting in increasingly more accurate works and, consequently, reducing the number of analog steps in the clinician's daily practice [21, 22]. Despite the simplicity of the various stages of treatment, digital workflow requires a steep learning curve as well as an understanding of the complications that may occur, such as deviations in implant position and intraoral scanning inaccuracies [13, 14].

Experience in digital treatment begins with planning. Software programs used in implantology for virtual planning require some steps that are essential for clinical success, including segmentation, deletion of artifacts, superimposition of images (DICOM/STL), and virtual implant placement [13]. Another important factor is the type of printer with which the virtual surgical guide is to be printed. The accuracy of surgical guides has been shown to be strongly associated with both the printing device and printing method [23]. Therefore, it is clear that there are many subtle points at the stages of planning and manufacturing the guide, in which the sum of errors may lead to disastrous results [13, 24].

Regarding the surgical procedure, guided implant surgery is generally faster than conventional freehand surgery and results in greater comfort for the patient in the postoperative period [25, 26]. Similar findings were observed in the present case report, in which guided surgery was performed without any complications, and the patient reported no pain or discomfort in the postoperative appointment. Although a guided surgery is mostly indicated in cases of flapless surgical implant placement [27], in our case, we opted for a guided surgery technique due to the limitations of the case. In bordering regions, such as the one reported here, there is a high risk of failure both in the surgical procedure, due to the extensive bone defect, and in aesthetic terms, due to possible gingival recession. In these cases, guided implant surgery is a favorable alternative to achieve the necessary precision for implant placement [4].

The precision of the guided surgery technique has already been demonstrated in the literature [28]; however, there are deviations that must be taken into consideration when developing virtual planning [13, 29]. In the current case report, we observed a deviation of 0.93 mm at the entry point, 2.2 mm at the apex of the implant, and an angular deviation of 5.5°, results different from those reported in a systematic review by Tahmaseb et al. [29], who observed a mean of 1.3 mm at the entry point, 1.2 mm at the apex of the implant, and 3.3° of angular deviation (Table 1). Although the deviation at the entry point in this case report was less than the average reported in the literature [29], apical and angular deviations were greater. These data alert us to the care needed in virtual planning, in the preparation of the surgical guide and in its printing, since there was no surgical complication. This can be considered a confounding factor in our study because, due to the several thorough steps of digital workflow prior to the surgical procedure, we were unable to understand what might have caused the observed deviations.

**Figure 11:**
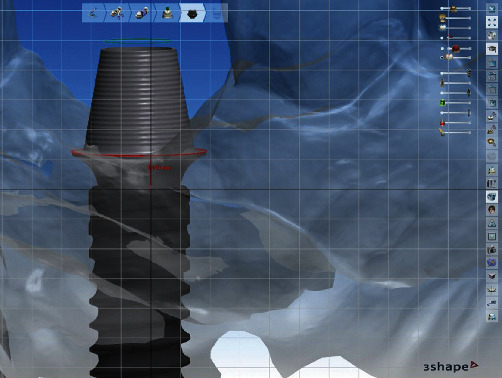
Evaluation of the abutment selected prior to the surgical procedure, demonstrating the accuracy of the technique.

Another important stage in digital workflow is prosthesis fabrication. In the present case, we opted for intraoral scanning to digitize the implant. Advantages included greater comfort for the patient and the possibility of clearly assessing the details of the scanned object, which allowed us to rescan any part that was not clear [14]. Obtaining a digital model also brings many benefits, such as not occupying any physical space and posing no risk of fracture. A great efficiency of the digital model, which we noticed in our case, was the ease of sending it to more experienced peers to exchange information about planning [30]. Evaluation of the final abutment in the virtual model (Figure 11) showed that our selection of abutment prior to surgical planning was accurate, leading us to believe that the observed deviations were not detrimental to the success of the final rehabilitation.

Given the precision of intraoral scanning [31], we decided to request the laboratory to send us the final crown, thus obviating the need for metal framework try-in. To this end, the final crown shade was selected using the intraoral scanner, and observed differences were compared to the VITA Classical shade guide. These results are compatible with those reported in the literature suggesting not to use intraoral scanning as the first option for color selection [32]. In our case, we sent the results of the two selections made (digitally and manually) to the laboratory for greater precision in the fabrication of the prosthesis. At the time of implant placement, only minor adjustments were needed, which indicates that digital workflow can simplify the clinical steps [21]. These data support the patient's positive assessment of treatment outcome. The patient reported that the procedures were not painful, especially the surgical procedure, for which she had great expectation of success. Also, she was comfortable in the postoperative period, and the prosthetic appointments were rapid and objective, meeting the patient's aesthetic and functional expectations.

## 4. Conclusion

Digital workflow is a reality in dentistry and can be integrated into daily dental practice. The clinical case reported here enabled us to demonstrate that virtual planning is safe, leading to faster clinical procedures with simplification of clinical steps as well as to a more predictable final result. It is important to note that there are still inaccuracies in digital tools, which must always be taken into consideration. We highlight that there is a learning curve for using these techniques, in which the sum of errors throughout the process may result in treatment failure.

## Figures and Tables

**Table 1:** Comparison between the deviations presented in the current case report and the mean deviations in partially edentulous patients reported in a systematic review by Tahmaseb et al. [29].

Deviations	Entry point (measured in mm)	Apex (measured in mm)	Angular deviation (measured in degrees)
Current case report	0.93 mm	2.2 mm	5.5°
Tahmaseb et al. [29]	1.3 mm	1.2 mm	3.3°
